# Understanding dual career views of European university athletes: The more than gold project focus groups

**DOI:** 10.1371/journal.pone.0264175

**Published:** 2022-02-25

**Authors:** Laura Capranica, Mojca Doupona, Ilvis Abelkalns, Ugis Bisenieks, Antonio Sánchez-Pato, Francisco José Cánovas-Alvarez, António J. Figueiredo, Juan Alfonso García-Roca, Alejandro Leiva-Arcas, Lourdes Meroño, Anda Paegle, Liliana-Elisabeta Radu, Cristian-Mihail Rus, Oana-Mihaela Rusu, Hugo Sarmento, Janis Stonis, Raquel Vaquero-Cristóbal, Vasco Vaz, Barbara Ghinassi, Pascal Izzicupo, Angela Di Baldassarre

**Affiliations:** 1 Department of Movement, Human and Health Sciences, University of Rome Foro Italico, Rome, Italy; 2 European Athlete as Student Network, Ghaxaq, Malta; 3 Department of Sport Sociology and History, Faculty of Sports, University of Ljubljana, Ljubljana, Slovenia; 4 University of Latvia, Riga, Latvia; 5 Olympic Studies Center, Faculty of Sport, Universidad Católica San Antonio, Murcia, Spain; 6 Research Unit for Sport and Physical Activity (CIDAF), Faculty of Sport Sciences and Physical Education, University of Coimbra, Coimbra, Portugal; 7 Faculty of Physical Education and Sport, University "Alexandru Ioan Cuza" of Iași, Iași, Romania; 8 Faculty of Sport, Injury Prevention in Sport Research Group, UCAM, Murcia, Spain; 9 Department of Medicine and Aging Sciences, University “G. D’Annunzio” of Chieti-Pescara, Chieti, Italy; Polytechnic Institute of Coimbra: Instituto Politecnico de Coimbra, PORTUGAL

## Abstract

Previous studies have found that student-athletes (S-As) have difficulties in achieving dual career (DC) success. However, no studies have analysed the opinion of the S-As on the functioning of DC with a qualitative methodology. The aim of the present work was to collect the opinions of elite university S-As in relation to DC policy adopted by their academic institutions in different European countries. In total, 77 athletes (F = 35, M = 42; age range: 20–25 years) participated in 15 national face-to-face focus groups in five different countries, to discuss aspects that higher education institutes should implement in relation to: 1) the athletes’ needs; 2) assistance/tutorship: 2) curricula requirements; 3) financial support; 4) logistic support; 5) social support; and 6) dual career policies. Fifty of the athletes competed in individual sports and twenty-seven team sports. Of them, 57 was enrolled at undergraduate, 17 was enrolled in a master and 3 in a PhD. The athletes were presented with 13 open-ended questions one by one, and were ensured freedom to interact. All the discussions were recorded. After this, a general discussion took place in which the participants identified and agreed on a final list of statements from their focus group deemed to be relevant to DC athletes as university students. Then, at a consensus meeting, the findings were combined, repetitions were eliminated, and fragmented statements were condensed into broader ones. A final list of 31 statements, organized in six related content units, were identified in relation to the athletes’ needs (n = 5), assistance/tutorship (n = 5), curricula requirements (n = 4), financial support (n = 4), logistic support (n = 4), social support (n = 6), and DC policies (n = 3), respectively. In conclusion, this cross-national qualitative research study synthesized the S-As views about their needs and the most relevant DC policies and provisions that higher education institutes should provide to ensure them with positive academic experiences towards the achievement of a degree.

## Introduction

The holistic development of elite athletes is considered one of the priorities, strategies and policies of sports around the word. Specifically, the European Union (EU), a geopolitical entity composed of 27 countries, has tried to turn into law, coordinate treaties, and manage a common budget between these countries to reach the all-round development of elite athletes [1–4]. However, due to cultural and organizational aspects, there is a limited relationship in many countries of the EU between the sport bodies and the educational institutions, which challenges student-athletes (S-As) to combine their academic and sport careers. As a consequence, they are at risk of academic or sports dropouts when considering professional sports and education as distinct and separate careers [5–7].

In recognizing the athlete’s right to combine sports and education with strategies such as the dual career (DC), some guidelines on DC of athletes have been published to promote and carry out actions in support of the holistic development of talented and elite S-As. For example, we find the guideline published by the European Commission [2], or more specific guidelines focusing on minimum quality requirements for DC services [6] and on qualifications/DCs in sports [8].

Specifically for higher education institutions (HEIs), the European DC guidelines call for a structured support to prevent elite athletes from engaging in personal negotiations with the academic staff to secure their educational path [2]. Furthermore, educational institutions must provide DC services with a minimum of quality, encompassing the inclusion of DC in the institution’s vision, strategy and policy; flexible educational programmes, and examinations for athletes through blended and distance learning; tutors and counsellors with a sound knowledge of DC challenges and opportunities; recognition of informal learning educational credits gathered throughout sports participation; availability of educational and sports facilities located within a reasonable distance; accommodation on campus; and plenty of visibility on traditional media and social media [2, 6–8].

Another of the strategies followed in the promotion of the DC has been the call for funds for investigating or promoting the links between the sports and education sectors at local, regional, national and transnational levels. For example, the European community Action Scheme for Mobility of University Students (ERASMUS+ Sport) programme, through which the EU’s sports policy is mainly implemented. This kind of funding program has tried to promote ethical and sustainable practices for developing a harmonized sport and academic environment, effective in the nurturing talented and elite athletes while respecting their educational needs [9, 10].

However, one of the major difficulties encountered in the implementation of the recommendations contained in these good practice guidelines is the differences in education and sport laws, structure and competencies between countries. In the specific case of the EU, competences in the field of education and sports mainly lie within the 27 member states. However, to support, coordinate, or supplement the actions of the Member States, a coherent approach to exploit the educational potential of sport is centralised in a common policy [11, 12]. Along this line, the EU designed a common European Higher Education Area which tried to homogenize the processes of accreditation and duration of degrees, curricula content, credit transfer and accumulation system, teaching methods, service provision, and organization of the academic year [13]. However, it was found that significant inter- and intra-country diversity existed, mainly due to national and local interpretations [14, 15]. Thus, a wide range of similarities and differences, both within and between European countries at higher education levels are still present [16].

The same applies to sports policies and organisations in the EU member states, with differences found in the sporting policy from the member countries, with local, national, regional and international sport bodies having their own rules and administrative organizations. In fact, The EU has a very limited direct role in sports although it recognizes the benefits of sports on personal and social well-being, its role in society, and its economic importance [17].

As a consequence of the above, the implementation of a homogeneous DC program for athletes in the EU member states has raised two important questions about the inherent complexity of DC policies and policy transfer: 1) member states adopt different DC policies, ranging from structured national legislations, formal agreements or sports bodies mediating with educational bodies, to *laissez-faire*/no formal DC procedures in place [5]; 2) there are different DC development environments in the European countries such as a) sports-friendly schools, (b) elite sport schools/colleges, (c) professional and/or private club programs, (d) sport-friendly universities, (e) combined DC systems, (f) national sports programs, (g) defines force programs and (h) players’ union programs with a range of approaches to supporting DCs [18]; and 3) the lack of a homogeneous definition of a DC athlete to equate the status of S-As in different member countries [6–8, 19]. Thus, in order to prevent the professional integration of athletes from being compromised by their sporting career, it is urgent to reduce the unfair exploitation of athletes’ rights in terms of access to and support for education and training.

To this end, in recent years, research into the topic of DCs in European countries has increased exponentially [20–22]. Along this line, some studies have been carried out that have analysed the perception of DC, highlighting challenges, significant stakeholders, and support programs with qualitative methods (e.g., interviews, focus groups, surveys) from the parent’s point of view [23] or with a global sample which included athletes and stakeholders [24]. However, only some studies have focused only in athletes from the same country and with a quantitative methodology [25, 26]. Whilst quantitative methods identify the extent of a phenomenon, qualitative methods allow us to understand the nature of the differences through the close contact, interaction and openness of the participants involved in the study [27, 28].

Therefore, although previous reviews have already pointed to the need to carry out studies that include S-As from different EU countries in order to obtain a global perspective of the difficulties encountered by these athletes in the development of their DC following a qualitative methodology [20], no research has been found that focus on this topic. Thus, the aim of the present study was to gather the opinions of elite university S-As in relation to the extant DC policy adopted by their academic institutions in different European countries.

## Methods

### Experimental approach to the problem

All procedures involving human participants performed in this study were in accordance with the ethical standards of the institution and the 1964 Helsinki declaration and its later amendments or comparable ethical standards.

The present work involved experts in virtue of their training or expertise. An expert was defined as a person who had information and knowledge in a substantive area beyond that of the average person and who regularly shared this information and knowledge through consultation, teaching or public speaking, or publications and written reports. For the Committee of the Protection of Human Subject (CPHS) purposes, experts are not human subjects when asked to provide opinions within their areas of expertise and do not require CPHS approval. Furthermore, in the present work, the experts’ opinion were about the external topic (e.g., factors deemed relevant for DC policies and services), not including demographic queries about income or other personal information. Furthermore, according with previous studies, this methodology allows describing the participants’ subjective viewpoints and experiences, such as their intentions, hopes, concerns, feelings and beliefs [29].

Participation in the study was voluntary and a signed informed consent was necessary to be included in the face-to face-focus groups. The participating experts were free to opt out at any time without providing any reason, and incomplete opinions were not considered. In the quest to ensure better knowledge on the DC of athletes at the HEI level, the rights and welfare of research participants were protected at all times, and confidentiality was ensured and maintained throughout the research study. For the above reasons, an IRB review was not required.

The assumptions that framed this research mainly followed an eminence-based approach that provided insight into four main themes: 1) the university S-As needs and expectations; 2) the current and envisaged academic eligibility of DC athletes; 3) the current and envisaged academic support and services for DC athletes; 4) the role of a DC and peer tutors. In exploring the experiences of university S-As, the research design was consistent with a critical realist epistemology, which allows gaining some insights into what is occurring and a good understanding of a context from the personal experiences and perspectives of individuals knowledgeable in the area under investigation [30, 31].

To gather the different S-As’ opinions and perspectives on DC at European HEIs, five European universities were selected on the basis of their extant DC policy adopted in relation to national DC policies presenting State-centric regulations (University of Coimbra, Portugal; and UCAM Catholic University of Murcia, Spain), State as sponsor/facilitator (University of Latvia, Latvia) and *laissez-faire*-no formal structure (University “G. D’Annunzio” of Chieti-Pescara, Italy; and University "Alexandru Ioan Cuza" of Iași, Romania).

In considering that a large number and broad composition of focus groups increases the representativeness and validity of the findings, several focus groups of 5–7 high-performance S-As were deemed appropriate to ensure the greatest amount of information gathered from group dynamics, a universal participation, and positive interaction between participants [32]. Thus, focus groups with dual-career athletes were conducted to stimulate collective discussions for building upon and questioning ideas and to reach a desired consensus on a series of statements describing their issues, problems, concerns and needs for the implementation of DC support at HEIs [33].

According to the literature [34, 35], the qualitative excellence of this study was guaranteed by means of: 1) external approval of the European committee; 2) the worthy topic based on DC research [6, 8, 22, 36, 37]; 3) coherence based on the development of More Than Gold guidelines for National focus groups with DC athletes as university student encompassing the research questions, the recruitment procedures of participants, the standard operating procedures and the instructions to be provided to the participants during the focus groups, and the data collection and synthesis; 4) the sincerity between the members of the research team in designing questions, in guiding focus groups without interfering with the participants’ opinions, and in analysing data without a pre-established attitude; 5) the credibility in fostering different perspectives to be mirrored in the outcomes of the focus groups; 6) the resonance grounded on the involvement of elite DC athletes enrolled in higher education courses; 7) a significant contribution of the findings as precious insights for developing DC at higher education institutes; and 8) the observation of fundamental ethical principles of the study regarding the benefit, fairness and awareness and anonymity of the participants involved in the study with encrypted information.

### Participants

To ensure the proper representation of athletes, a purposeful sampling technique was deemed appropriate and a core strength for gaining comprehensive, meaningful, and practical knowledge [32]. Thus, the following inclusion criteria were considered: 1) competitive level (e.g., athletes competing at the Olympic Games, World and European Championships, University, international Cups, and national level competitions); 2) type of sports (e.g., individual and team sports); and 3) academic level (e.g., undergraduate and graduate). In total, 77 athletes (F = 35, M = 42; Italian: n = 17; Latvian: n = 17; Portuguese: n = 15; Romanian: n = 15; Spanish: n = 13) ranging in age from 20 and 25 years old, competing in individual (n = 50; athletics, karate, judo, skeleton, triathlon) and team (n = 27; field hockey, hockey, soccer, rugby, volleyball) sports and enrolled in undergraduate (n = 57) and graduate (n = 20; master’s: n = 17, PhD; n = 3) academic levels in different majors in all the European Research Council areas (e.g., Social Sciences and Humanities, Physical Sciences and Engineering, and Life Sciences) provided their written consent to participate in 15 national face-to-face focus groups, each lasting an average of 2:30 hr:min, from January 7^th^, 2020 to March 1^st^, 2020.

### Procedures

At the beginning of the face-to-face focus groups, the organizers provided a 10 min presentation on the MTG project, its aims, the expected participants’ contribution established in an open-ended and non-judgmental fashion, and the operating procedures of the focus group. In addition, they were provided with a glossary of concepts and definitions of some of the terms (e.g. sport tutor or DC tutor; student-tutor or peer-tutor) to be covered in the focus group in order to homogenize the nomenclature used in the focus groups. Along this line, the participants were provided with the 13 questions to be addressed in an ordered sequence (Table 1). The questions were developed not just to document the participants’ experiences in the subjective sense, but also to explain life trajectories [29].

**Table 1:** Sequence of thirteen questions addressed during the national focus groups and overall outcome statements to guide the development and/or implementation of dual career (DC) academic support and services at Higher Educational Institutions (HEIs).

Sequence	Questions	Statements
		(total n = 408)
1	In your opinion, what does “High performance Athlete” mean?	38
2	How easy/difficult is it for you to balance your sporting life with your academic life?	38
3	Who supports you towards achieving a good balance between your sporting life and your studies?	46
4	What are your expectations for the end of your studies?	31
5	What kind of technological or teaching tools do your teachers use to ease your progress in class?	21
6	What aspect/s of the University would you change to ease your DC?	45
7	What is the most difficult aspect of your athlete career for you to coordinate with your studies?	44
8	How highly do you value these services and features of the DC at your university?	22
9	With which aspect would you like your sports tutor to advise/help you?	32
10	What is for you the best way to be in touch with your ‘DC Tutor’ or ‘Sport Tutor’ and how many times per month?	31
11	How does the ‘DC Tutor’ or ‘Sport Tutor’ help you?	14
12	How does the ‘student-tutor’ or ‘peer-tutor’ help you?	20
13	What else would you like to say?	26

DC: Dual career.

Whilst a researcher acted as a facilitator, another manually registered potential supplementary observational data (e.g., context, personal gesture, posture, etc.). The athletes were presented an open-ended question and were ensured freedom to interact directly, by sharing personal experiences and personal anecdotes, questioning one another, building upon one another’s views, agreeing or disagreeing with opinions. Each athlete was encouraged to write the most important factors considered relevant for answering the question on a flipchart. To establish trustworthiness, member checks have been used for establishing credibility [38, 39]. Thus, when all the 13 questions had been addressed, a 20/30-minute general discussion took place with all the participants to identify and agree upon a final list of statements from their focus group deemed relevant to DC athletes as university students. This final consensus was deemed relevant for establishing the validity and reliability of the statements.

### Data analysis

To analyse the focus group data derived from multiple focus groups carried out on the same general topic in different countries, and to maintain consistency with the original purpose of the research, for each country two bilingual members of the research team, whose mother tongue was the native language of the country where the focus group was held, independently translated and contextualized into English, in terms of the cultural aspects of the country where it was carried out, any statement (e.g., word, short phrase or sentence) recorded during the National focus groups. Afterwards, they agreed on a combined version, which was subsequently backward translated by an English reviewer using a blind translation procedure [23]. Then, at a consensus meeting of 21 DC experts of the MTG project, the findings were combined, repetitions were eliminated, and fragmented statements were condensed into broader ones. To establish trustworthiness, a debriefing session took place to analyse all meaning units, themes, and categories [38, 39]. Thus, an inductive thematic analysis was deemed necessary for organizing and interpreting the recorded statements into content units [40, 41].

## Results and discussion

### Content units stablished by DC athletes

A total of 408 descriptive statements of DC athletes were analysed and synthesized into a final list of 31 statements, organized into seven related content units (Table 2): student-athlete, assistance/tutorship, curricula requirements, financial support, logistics, social support and polities. Previous studies which investigated the needs of the athletes involved in DC with a qualitative methodology from the athlete’s parents point of view [23], and a mix of athletes, coaches, sport and DC support staff, parents, health team, and teachers [24] point of views, found similar dimensions which referred to the athlete’s needs, support team, assistance/tutorship, curricula requirements, logistics, social support, and policies and services [23, 24]. Unlike previous studies, the athletes in the present research identified funding as a key unit, which had not been identified in previous research [23, 24]. Bearing in mind that in most cases the scholarships these athletes received were dependent on their sporting performance [7, 19], and it is possible that they are aware that they are gaining access to a number of advantages as athletes that they would not otherwise have.

**Table 2:** List of statements to guide the development and/or implementation of dual career (DC) academic support and services Higher Educational Institutions (HEIs) for student-athletes (S-As).

Content Units	Statements	Philosophy	Examples
Student-athlete	DC eligibility for talented and elite athletes	S-As status for both elite athletes and athletes committed to organized sport within their respective sport federations and engaged in demanding training and competitions independently from the competition level	“The one who spends most of his time doing a sport whose goal is to reach the highest level”
			“It is not necessary to be selected by the national team to be eligible”
	Support from the athlete’s personal entourage (family, peers)	Relevant supportive roles of family (parents, siblings, couple) and friends	“I support myself in the people around me, but the most essential are my family and closest friends”
	Support from the athlete’s sport entourage (coach, manager, federation)	Relevant supportive roles of coaches, sports directive, institutions, partners	“Very important pillars and they are always there of course, my team, coach and players that in the end become a second family, I am nothing without the support from all of them, because they trust me and that gives more confidence in myself”
	Support athlete’s from the academic entourage (teacher, classmate, DC tutor)	Relevant supportive roles of teachers, classmate, DC tutor	"The support from the DC Tutor and teachers was awesome"
			“My university has been an important source of support”
			“My classmates help me a lot. When I can’t go to class, I get a lot of help.
	Professional expectations	S-As have high professional expectations for their sports career but they were aware that their competitive years would naturally come to an end and about the importance of higher education to improve their chances for future employment	"To have a training session and to be able to live from the sport"
			“I hope to finish my sport career and after to be able to dedicate myself to what I have studied”
Assistance/tutorship	Adaptable DC programmes (based on specific contexts)	Specific and flexible DC programmes, conceivably capable to anticipate the needs of S-As	“Be a little more flexible with the exam changes, that even if you arrive at the exam date, understand that you have been competing for the whole previous week outside of my country and that you have not had time to study. I think that is a reason enough to be able to modify the date of an exam.
			“Our competitions coincide with weekends, which is when people study and do their tasks”
	Integrated DC programmes (based on intra- and inter-departmental cooperation and professional services)	To overcome the existing fragmentation of DC support structures and to at least partially resolve the lack of dedicated DC programmes or services, intra- and inter-departmental cooperation was foreseen	“It is necessary to establish an athlete’s office at the university. From here we could facilitate the follow-up of the dual career by establishing more clear and detailed programs for the follow-up of the dual career. At present, not all departments and professors act in the same way”
	Proactive DC programmes (capable to act autonomously, even anticipating needs)	To train student service personnel with topics relevant for athletes	“There are teachers who are not related with sports and do not understand our situation"
			“I would like that the sport tutor or the athlete’s office teach professors and staff about the needs of S-As and how to help them”
	Psycho-social support	HEIs may provide services to enhance the student’s experience and to secure graduate employment (e.g., academic orientation, consultation, career and personal counselling, nutrition, medical, and psychological support)	“Diet is a difficult aspect in my case. I practice a weight-class sports but there are times when I can’t get the right amount of food because of class schedules. It would be good to have counselling at the university in order to achieve balance in my DC”
			“We need psychological support”
			“It could be good to receive a physiotherapy treatment”
	Tutorship/mentorship (Specific DC methodologies)	Establish an informal relationship with a tutor/professor, also taking advantage of social means of communication considering on-line reception and support, and e-learning services	"Send an email and they answer you instantly, it’s awesome"
			"Thanks to these tools, you do not feel like you are not attending at class"
Curricula requirements	Distance learning	S-As’ need for distance learning, especially when they face conflicting schedules due to sport commitments	“Training schedules sometimes complicate study schedules”
			“The trips and the trainings are the most difficult aspect to coordinate athlete career and studies”
	Individualized study plan	S-As’ need for flexible curricula and individualized study plans	“S-As may have an extra time to send their work during the competition season"
			“I have a number of advantages that facilitate many things: Changes in exam dates and non-compulsory attendance to theoretical classes, among others”
	Recognition of the sport career for academic credits (ECTS)	Recognition of their competences and qualifications acquired through non-formal and informal sports education as a part of or in addition to their formal education	“Sports should be popularised. The practice of sport has many benefits and the University does not give them the necessary promotion and they are not included in the educational programmes. They seem to be secondary”
			“It is necessary to view and recognize the sport career in academic life”
	Untraditional learning strategies (e.g., creating digital portfolios, using social networks)	Assessment and use in the assignment of non-traditional learning strategies (e.g., creating digital portfolios, using social networks)	“To ease the DC, the demands of the exams need to change. I believe that studying for the exam is not the best way to learn. Use of other tools such as portfolios or tasks may allow better learning”
Financial support	Scholarships	Need of scholarships especially if engaged in non-revenue sports	"I am a privileged person, and I do not want to waste the opportunity that has been given to me, and that many colleagues have not been able to enjoy"
	Remission of tuition fees	Need of remission of tuition fees especially if engaged in non-revenue sports	“The university would need to change to ease the DC, by giving scholarships to S-As, especially when the athlete does not have a salary”
	Salary	Provide them a position of financial security especially if engaged in non-revenue sports	“The most difficult aspect of my DC is financial issues. There are a lot of expenses from both the academic and sporting side, plus living away from home”
	Others (e.g., sponsors)	A demanding financial burden of DC was reported when HEIs do not provide subsidy actions to sustain enrolment and living costs of athletes, especially in absence of financial support from sport clubs or federations to cover the costs for equipment, training camp, and travel	“We have a lot of travel and living expenses that are often not covered by anyone”
			“I need economic support from the University because I don’t have it from the sport”
Logistics	Access to educational facilities (e.g. gymnasium, internet, e-mail services, e-libraries, labs, research centres)	University services should be located near from the sport centre and properly conditioned	“To move from the sports centre to the campus is the most difficult aspect to coordinate my DC. I need accessible structures closer”
			“The University facilities should be more accessible for athletes on wheelchairs”
	Accommodation facilities for S-As	Implementation of the transport system and living near from study and training centre	“The balance between my sporting life and my academic life is extremely difficult due to transport. It is something that needs to be improved”
	Economic investment for university facilities	Necessity of economic investments for university facilities as sport	“There is a lack of accessibility to some university structures”
			“Sports facilities should be available for S-As”
			“We need free access to sport facilities at the university”
	Sport facilities	Construction of sports facilities within the HEI	“I don’t have a gym in the campus, so I need to move out”
			“The university should have its own stadium”
Social support	Institutional DC committee	Institutional DC committees for the dialogue between DC actors (e.g., teachers and academic staff, coaches, parents, students) could be established	“The DC office should let the coach/team director communicate with the sport tutor”
	Local to international seminars, workshops, and meetings on up-to-date DC issues	Information on DC issues could be disseminated by means of seminars, workshops, and meetings	“The university should organize events about dual career”
	Peer to peer support	HEI services facilitating friendships with non-athlete classmates with, for example, peer-to-peer tutoring	“My student tutor and several athletes have a Whatsapp group, and we are friends”
	Publicity for S-As representing university	HEIs could publicize their team or individual sports accomplishments	“I would establish a hall of fame for S-As in each university”
			“I think we can have an acknowledgment on the university web page”
	Publicity on the S-As and their characteristics suitable for the labour market	S-As can be valuable resources for their HEIs in fund-raising activities, promotion of events, representing their university in public events, or connecting with trends of the labour markets to establish virtuous cooperation also with the private sector	“At my university the most famous S-As are used to promote the university’s advertising, especially in recruitment campaigns.”
	Seminars, workshops, meetings with parents and coaches	S-As can be role models providing inspiration and publicity for the actual and prospective student body, attracting larger pools of qualified student bodies	“Having S-As at your university is a win-win for both parties. There is no better publicity to raise funds for the university than having well-known athletes”
Policies	National DC policies	Need of clear DC policies at national level, which are needed to establish a coherent framework for the combination of sports and education	“I would like that services for dual career will have regulations”
			“Changes in legislation are necessary, including at the faculty and university level”
	Special access contingent (reserved for actual or ex high sport performance practitioners)	Observatories could start collecting information regarding the sport participation (e.g., type of sport, competitive level, sports commitment) of university students, which could allow large-scale monitoring	“It would be easier if there was someone, a support centre, whom to trust and ask for a help to solve a variety of organizational issues both in studies and sports”
			“There is a need for the S-As service to be centralised to allow for a proper follow-up of S-As”
	Sport observatory of the university (controlling and monitoring the application of the DC statute)	At individual HEI, academic monitoring could also encompass direct consultation of the S-As or their DC tutors with faculty members to confront perspectives on strengths and weaknesses	“All universities and departments should hold such discussions to find out what is not working in their dual degree programmes and to improve them”

DC: Dual career; HEI-s: Higher education institutions; S-As: Student-Athletes.

#### Student-athlete content unit

In particular, five statements pertained to aspects related to the S-A were found (e.g., eligibility and support for DC paths, and professional expectations). The philosophy shown by the athletes in this section as well as some examples can be found in the Table 2. Specifically, by delving deeper into the content unit on the student-athlete, the participants of the focus groups envisaged the recognition of S-As status for both elite athletes and athletes committed to an organized sport within their respective sport federations, and engaged in demanding training and competitions independently from the competition level. In fact, both elite and sub-elite athletes engage in sports with demanding training and competition schedules, which challenge them to combine their DC paths, especially in the absence of academic and/or sport DC support, lack of flexible academic and sport schedules, and in the presence of long distances between the university and the sport venues [25, 26].

At present, the lack of a clear and universal definition of talented and elite athletes, as well as country-specific cultural and organizational regulations in the field of sports and education, determine important differences in the eligibility criteria and requirements for DC programmes of S-As [6, 8, 42], with the athletes who participated in the present investigation showing their concern about this. More specifically, it is common that eligibility for academic admission procedures and DC paths and services include a limited number of elite athletes who are members of national Olympic and Paralympic teams, and/or placed in the top positions of the World and European senior or junior championships in Olympic sports [8]. However, the construction of a sport career requires several years of deliberate practice, and top-level performances in some sports could be achieved in adult age [43, 44]. Due to all of the above, it would be desirable for different countries to adopt policies similar to the one recently adopted in Portugal, which defend the rights of S-As to achieve success in their DC from an early age, thus recognizing that the regular practice of sports activity in an academic context is an important complement to the holistic training of an individual as well as to the promotion of the development of healthy habits throughout life [45]. This is an important scope to address in the future.

In addition to their motivation, capability to juggle two commitments, to manage busy daily schedules, and to stay focused on both sport and education achievements, the S-As highlighted a crucial role of their support entourage. Independently from the provision of DC in their respective countries, relevant supportive roles from personal, sport and academic entourage were emphasized. These findings substantiate the literature on the DC challenges in different contexts and the importance to access a coherent career support network [22–24, 46, 47]. Indeed, the academic years often coincide with increased competitive demands and new personal adjustments due to academic responsibilities and changes in residence and distance from family and friends, and detachment from the student cohort, particularly during the first year at the university [48, 49]. For this, a collective effort is important, and regular communication across the athlete’s sport and education entourage were envisaged, also including more experienced S-As as role models [41, 49].

As for the professional expectations, despite the participants in this study declaring high professional expectations for their sports career, they were aware that their competitive years would naturally come to an end. Thus, they deemed higher education important to improve their chances of future employment, as they were also aware that the labour market favoured employees possessing a university degree [50]. According to the literature, independently from their short-term athletic goals, the S-As enrolled in a variety of university majors, based on their individual attitudes, motivation towards sports and academics, career planning, and the constrains of the degree programme which determined the study regime, forms and periods of examination [46, 51–56]. More specifically, in enrolling in educational paths, elite athletes could have a status of S-As, but the benefits offered by educational institutions are very different in regard to type and degree, generally related to reductions of compulsory attendance and other study obligations, the possibility of prolonging the length of the study programme, the opportunity of passing exams outside the regular deadlines, financial and logistic supports [57]. In fact, the adjustment of academic obligations of the S-As depends on the individual HEI within its national DC approach. More specifically to the HEIs included in this study, the Spanish (Catholic University of Murcia, UCAM) and Portuguese (University of Coimbra) have well-established DC programmes, encompassing a specialized tutoring system and DC services [37, 45], the University of Latvia is a sports-friendly higher education institution [58], whereas the University "Alexandru Ioan Cuza" of Iași, Romania, and the University “G. D’Annunzio” of Chieti-Pescara, Italy, did not consider the needs of the S-As as a talented atypical population [59, 60]. In fact, only some Italian HEIs have implemented their policies and services to support DC and/or have established agreements with national sports federations, so that DC interventions are restricted to a particular academic-sports environment and involve a limited number of elite athletes, whereas the DC in Romania is at its infancy level [59, 60]. Moving towards DC programmes that allow the athlete to be successful and to be able to combine their studies and sport is an important challenge for the future, regardless of the institution where they study.

#### Assistance/tutorship content unit

Five statements pertained to aspects related to the assistance/tutorship were found (e.g., DC programmes, tutoring, and psychosocial support) (Table 2). Despite the existing differences between the available DC supports, in this study the participants envisaged specific and flexible DC programmes, conceivably capable of anticipating the needs of S-As. To overcome the existing fragmentation of DC support structures and to at least partially resolve the lack of dedicated DC programmes or services, intra- and inter-departmental cooperation was foreseen. Considering that HEIs usually provide various services to enhance the non-athlete student’s experience and to secure graduate employment (e.g., academic orientation, consultation, career and personal counselling, nutrition, medical, and psychological support), the participants in this study suggested that HEIs deem appropriate the training of student service personnel with topics relevant to athletes, such as DC transitions and development, training for academic success, skills transfer, emotional distress, coping, time management, relaxation, and positive working relationships with coaches, athletic administrators, and academic staff. In fact, the success of these services may depend on the DC awareness at the level of their staff [6].

Following the publication of the EU Guidelines on DCs of Athletes, HEIs were urged to adopt new pedagogical models and processes, and to provide a tutoring system for their student athletes. In fact, European universities were challenged to invest on new ways of reorganizing knowledge, rules and learning models to offer sound education to their students [39, 61]. Consequently, several European projects focused on the development of a dedicated guidance and tailor-made arrangements to sustain S-As [9]. In the search for solutions to the challenges of educating athletes, a flexible educational model was developed, taking into account the differences between HEIs in curriculum structures, whereas several ERASMUS+ Sport Collaborative Partnerships focused on the competences of support providers, tutors or mentors [62, 63]. These interdisciplinary approaches contributed to the European DC discourse based on eminence, and evidenced research findings on DC support services and competences, which should foster the implementation of DC policies and provisions at the European HEI level [20, 22]. However, legal constraints and university administrative rules may not favour the introduction of DC programmes in some member states. In this regard, tutorship becomes crucial to help with the development of virtuous interactions between the different DC actors for the psychosocial development of the S-As [7, 19, 36]. For mediating with academic staff and teachers to find creative solution to their needs, the participants deemed relevant the establishment of an informal relationship with a tutor, also taking advantage of social means of communication. In fact, through personal negotiations, teachers could be available to provide additional work in case of missed class attendance, considering on-line reception and support, and e-learning services, as appropriate tools for athletes [19, 64].

#### Curricula requirements content unit

Another topic mentioned by the athletes was curricula requirements. In particular, four statements pertained to aspects related to curricula requirements were found (e.g., individualized DC paths and learning strategies) (Table 2). According to the European DC recommendations [2], the present findings corroborate the S-As’ need for flexible curricula, individualized study plans and distance learning, especially when they face conflicting schedules due to sport commitments. When available, S-As can take advantage of these elements during their academic path on a voluntary basis, and could also select only a few of these opportunities. Conversely, in countries with no DC policies in place or tied to traditional didactics, severe legal constraints and university administrative rules uphold face-to-face education, exacerbating the difficulties of S-As. However, during the recent COVID-19 pandemic lockdown, the European HEIs were confronted with a prompt and agile response to continue their academic activities, the adoption of remote methods [25, 65]. At present, distance learning is no longer a new concept, and HEIs developed sustainable e-learning systems including recorded lectures, which could be maintained to allow distance learning and academic flexibility, to guarantee future opportunities for an adequate support of S-As and other atypical students [66].

In Europe, a clear academic European Credit Transfer System (ECTS) procedures between HEIs in different EU Member States are deemed essential to the European Higher Education Area, and the development of new skills through a range of different experiences outside formal education is recognised to facilitate an employer or education institution with knowledge of the actual qualification of an individual [13]. Indeed, sports participation can advance competence-based approaches to educational outcomes in addition to the formal academic paths [8, 61]. During the focus groups, the participants envisaged the recognition of their competences and qualifications acquired through non-formal and informal sports education as a part of or in addition to their formal education. In fact, athletes learn valuable life lessons from their participation in sports, and former-athletes reported transfer of skills and attitudes from sports to their post-sports career, including energy, commitment, teamwork, respect, modesty, goal orientation and adjustment, mentoring receptiveness, self-efficacy, time management, responsibility, and autonomy [61, 67, 68]. Moreover, these skills can have positive effects on career success and labour market trajectories of former athletes, with higher salaries with respect to their non-athlete counterparts [69]. This is an important issue that needs to be addressed in the future by the different agencies involved.

#### Financial support content unit

Four statements pertained to aspects related to financial support were found (e.g., fees and salaries) (Table 2). One of the main novelties of this research was the inclusion of financial support as a main topic based on the opinions of S-As. According to the European education area, HEIs strongly contribute to knowledge production for the development of highly skilled human capital and the promotion of upward socio-economic growth, equal opportunities, health and life satisfaction. At present, European Member States have adopted different policies to cover the financial aspects of tertiary education, ranging from full state support when considering HEI as a public good, to high student tuition fees when HEI are viewed as a private investment of the student, thus adopting low-tuition–low-subsidy, low-tuition–high-subsidy, or high-tuition–high-subsidy regimes [70]. In this study, the sample included either athletes enrolled in low-tuition–low-subsidy or high-tuition–high-subsidy HEIs. In reporting that a limited number of S-As received financial support that payed for all or part of their expenses, the participants highlighted the need for remission of tuition fees, scholarships, and subsistence, to provide them with a position of financial security, especially if engaged in non-revenue sports. In fact, a demanding financial burden of DC was reported when HEIs did not provide subsidies to sustain enrolment and living costs of athletes, especially in the absence of financial support from sport clubs or federations to cover the costs for equipment, training camp, and travel, which are necessary for preparing to compete at the highest levels [23, 71, 72].

The financial pressures could be responsible for DC dropouts [73]. Whilst the student’s enrolment in a university path could be affected by the cost of higher education (e.g., tuition fees, books, housing and food, etc.), DC opportunities should also be considered when HEI’s brand matter [74]. In this respect, HEIs could consider DC as the basis of their brand-related vision and values grounded in sports and active lifestyles. Thus, implementing strategic processes that align internal and external dimensions could help HEIs achieve collaborative practices to co-create sport-related values with the S-As [75–77].

#### Logistics content unit

Logistics was also highlighted as a relevant topic. Specifically, four statements pertained to aspects related to financial support were found (e.g., academic and sport) (Table 2). According to the literature, the participants in this study declared that they wasted time when travelling from their home to the university and the sport venues [45]. To optimize their very busy daily schedules, the athletes envisaged the implementation of a transport system, as well as HEI housing, educational, and the sport facilities. At present, the European Study on Minimum Quality Requirements for DC Services claimed that the educational (e.g., classes, laboratories, study rooms equipped with information and communication technologies, e-libraries), accommodation (e.g., sleeping facilities, cafeterias) and high performance training centres (e.g., strength & conditioning gyms, recovery and therapy rooms, training venues) should be located within a maximum of 15 minutes travel time [6].

The participants in the focus groups also envisaged economic investments for university facilities. In some countries, the logistic aspect for athletes is considered very relevant, and a rapid build-up of HEI facilities has been reported, with the objective of attracting new students-athletes to campus [78]. However, no country prizes university sports as the United States. European S-As who do not have DC opportunities in their home countries tend to enroll in American HEIs, which offer sport scholarships, on-campus living and sports facilities, academic flexibility, distance learning, support resources, counselling, and well-structured DC paths [79–81]. To limit trans-continental migration of S-As, and to enhance the European DC, Member States should consider the implementation of HEI facilities for S-As and transnational dual-career cooperation to increase the mobility of S-As within Europe.

#### Social support content unit

Six statements pertained to aspects related to the social support were found (e.g., supportive entourage and public recognition) (Table 2). According to the literature, the present findings substantiate the athlete’s feeling of a limited social life aside from training and study [46]. To have meaningful lifetime experiences as university students, relationships with academic faculty and non-athlete peers could be enhanced by HEI services facilitating mentorship from professors and friendships with non-athlete classmates [19, 82, 83]. Thus, institutional DC committees for the dialogue between DC actors (e.g., teachers and academic staff, coaches, parents, students) could be established, and information on DC issues could be disseminated by means of seminars, workshops, and meetings [84]. When considering the relevant role of non-athlete classmates in helping S-As connect with the academic life and forming friendships outside of their athletic circle, HEIs could foster peer-to-peer tutoring. Especially for the first-year S-As in need to adapt to a new environment, peer mentoring is a relevant aspect of effective undergraduate education, and it could help increase the sharing of information from classmates and/or other S-As with an advanced DC path, who are academically and socially connected, thereby providing opportunities for social interactions, academic engagement and connectivity, and for avoiding academic dropout [85]. On the other hand, due to their role as consistent and immediate references for the S-As, peer mentors could experience leadership opportunities, which could be promoted through ECTS recognition and economic support.

To increase the S-As’ sense of belonging and to avoid their isolation and distancing from their non-athlete peers, HEIs could publicize their team or individual sports accomplishments. Despite European university sports usually not bringing in revenues, S-As can be valuable resources for their HEIs as role models, by providing inspiration and publicity for the current and prospective student body, fund-raising activities, promotion of events, representing their university in public events, or attracting larger pools of qualified student bodies. In fact, the athletes’ actions inside and outside sports do not go unnoticed, and could provide benefits to their campuses, society, and the sports sector, also through their social media network [86]. Within the HEIs’ autonomy, some universities encompass financial compensation to their S-As [87]. Unfortunately, most of the European HEIs do not value their S-As, having no awareness of their number and/or offering no forms of recognition or usage of their image [6]. Finally, HEIs should consider providing proper publicity on the S-As’ characteristics as valuable human resources, for connecting with trends in the labour markets and to also establish virtuous cooperation with the private sector.

#### Policies content unit

Three statements pertained to aspects related to the policies were found (e.g., national and local recommendations and monitoring systems) (Table 2). The findings of the present study also highlighted the need for clear DC policies at the national level, which are needed for establishing a coherent framework for the combination of sports and education. Despite the European Commission urging Member States to develop national DC guidelines, at present only Sweden has published a policy document resulting from the cooperation between DC stakeholders, including researchers and the research users [2, 88]. Whilst national laws and regulations are available in Spain and Portugal, in Italy the recently-established Italian network of universities interested in sports (e.g., UNISPORT Italia) started to provide information on which universities offer DC programmes for athletes, and fostered the cooperation between the Italian Conference of Rectors with the National Olympic and Paralympic Committees. Indeed, these actions substantiate the European efforts to support tertiary education of S-As.

At present, no monitoring system to assess DC responsibilities, programmes and provisions at local, national and European levels has been adopted, which limits the possibility for objectively evaluating the DC phenomenon and to take action in support of S-As, when necessary [6]. Considering that governments have established observatories for gathering a comprehensive overview of HEIs’ programmes, services, trends, and students’ progresses, it is possible to envisage their implementation with respect to the student-athlete subgroup. Thus, these observatories could start collecting information regarding the sports participation (e.g., type of sport, competitive level, sports commitment) of university students, which could allow large-scale monitoring. At individual HEIs, academic monitoring could also encompass direct consultation of the S-As or their DC tutors with faculty members to obtain their perspectives on strengths and weaknesses, so that actions for improving academic performances can be also planned according to the constraints of the competitive season.

### Practical applications of this study

The present qualitative approach based on the experiences and visions of European S-As enrolled at universities with different DC policies, if any, led to the identification of areas for implementation of DC policies and provisions that are sufficiently specific and generalizable. The findings of this exploratory study setting the foundation of knowledge about the quality implementation of the DC at the European HEI level.

### Limitations and future lines of the current study

Some main limitations are still present. First, the highlighted aspects could be interconnected and may not stand on their own. In fact, the DC is a multi-faceted process, which needs to be managed and framed within different contexts and cultures [8, 22]. Therefore, future research should be focused on specific ways in which these aspects relate and mediate to improve DC tertiary educational programme development. Secondly, the relevant information pertaining to the DC at tertiary education could not be determined solely from analysing the S-As views and expectations. Thus, the S-As’ views need to be integrated with those of HEI experts, who could assess the relevance and feasibility of the implementation of the proposed aspects in terms of quality measures. When involving HEI experts with varying backgrounds and roles (e.g., faculties, managers, psychologists, DC tutors), future qualitative and quantitative research could provide in-depth analyses of a wide range of complex policy questions and expand our knowledge on alternative objectives for creating strategies to efficiently allocate resources between different options. Altogether, this knowledge could have implication for practitioners in the application of deliberate strategies to produce continuous improvements that could guarantee the positive academic experiences university S-As, and provide effective support towards the achievement of a university degree. Thirdly, as this research focuses on university DCs and not on athletes still in school or working, it would be necessary to contrast the results from the other vital moments of the student-athlete in order to obtain a global and complete perspective about their difficulties in reconciling their different performances in the different stages of their lives [22]. Finally, the athletes’ difficulties in achieving DC success may vary depending on the legislative and institutional support they have, as previous research has found [5, 8]. Therefore, the extrapolation of these results to other countries or institutions should be made with caution.

## Conclusions

This cross-national qualitative research synthesized the athletes’ perspectives about their needs as university students. The present findings highlighted six key DC aspects, which are often interconnected. Furthermore, they not only represent the basis for the development of guidelines for the implementation of DC at European HEI level but also have a high potential applicability and generalizability for real changes.
